# Predictors of male circumcision incidence in a traditionally non-circumcising South African population-based cohort

**DOI:** 10.1371/journal.pone.0209172

**Published:** 2018-12-19

**Authors:** Katrina F. Ortblad, Till Bärnighausen, Natsayi Chimbindi, Samuel H. Masters, Joshua A. Salomon, Guy Harling

**Affiliations:** 1 Department of Global Health, University of Washington, Seattle, Washington, United States of America; 2 Department of Global Health and Population, Harvard T.H. Chan School of Public Health, Boston, Massachusetts, United States of America; 3 Africa Health Research Institute, Mtubatuba, South Africa; 4 Institute of Public Health, Faculty of Medicine, Heidelberg University, Heidelberg, Germany; 5 Institute for Global Health, University College London, London, United Kingdom; 6 RTI International, Research Triangle Park, North Carolina, United States of America; 7 Department of Medicine, Stanford University, Stanford, California, United States of America; 8 Institute for Global Health, University College London, London, United Kingdom; NPMS-HHC CIC / LSH&TM, UNITED KINGDOM

## Abstract

**Background:**

Voluntary medical male circumcision has been promoted in high HIV prevalence settings to prevent HIV acquisition in males. However, the uptake of circumcision in many sub-Saharan African settings remains low. While many studies have measured circumcision prevalence, understanding circumcision incidence and its predictors is vital to achieving ambitious circumcision prevalence targets.

**Setting:**

Rural KwaZulu-Natal, South Africa.

**Methods:**

We measured circumcision incidence over the period 2009–2014 in a longitudinal population-based cohort with high HIV prevalence and low circumcision prevalence. Multivariable survival models with Weibull distributions were used to assess socio-demographic, behavioral and biological predictors of circumcision incidence.

**Results:**

Between 2009 and 2014, circumcision prevalence among males 15–49 years in the cohort increased from 3% to 24%. Among 6,203 males 15–49 years, 873 new circumcisions occurred over 13,678 person-years (incidence rate: 6.4/100 person-years, 95% CI 6.0–6.8). Circumcision incidence was substantially higher amongst young males: 15–19 year olds were twice as likely to circumcise as older males. In the survival model, shorter household distance to the nearest healthcare facility, knowledge of HIV status and biological HIV-negative status were associated with an increased likelihood of circumcision incidence.

**Conclusions:**

Circumcision prevalence among males in rural KwaZulu-Natal remains well below South Africa’s national 80% coverage target across age groups. In this population, distance to the nearest healthcare facility and knowledge of HIV status were important independent predictors of circumcision incidence. Mobile circumcision clinics and innovative HIV testing services may be important tools to help achieve circumcision targets.

## Introduction

Global voluntary medical male circumcision (VMMC) prevalence and incidence remains low despite efforts to scale the HIV prevention intervention.[1,2] VMMC is one of the most effective HIV prevention interventions; three randomized controlled trials found that it reduced the risk of female-to-male HIV transmission by approximately 60%.[3–6] Since then, VMMC has been included as a key component of combination HIV prevention strategies, which haven successfully shown reductions in population-level HIV incidence.[7,8] One of the advantages of VMMC compared to other HIV prevention interventions, such as condom use or pre-exposure prophylaxis, is that its effectiveness does not rely on repeated and consistent behaviors. In 2010, the World Health Organization (WHO) recommended VMMC as an important strategy for the prevention of HIV transmission and identified 14 priority countries in eastern and southern sub-Saharan Africa for targeted scale-up of VMMC.[9,10]

South Africa, one of WHO’s 14 VMMC priority countries, [2] began expanding VMMC programs around 2010 and set ambitious national and provincial targets to achieve 80% circumcision coverage among males 15–49 years by 2015, and maintain this coverage through to 2025.[9,11] South Africa’s VMMC guidelines encourage all males not living with HIV to undergo VMMC at their nearest health facility. Per the guidelines, males living with HIV can still undergo VMMC, but they should be advised that this will not reduce their risk of transmitting HIV to their sexual partners.[12–14] To achieve national VMMC targets, South Africa has employed communication campaigns (e.g. mass media, billboards, interpersonal recruitment), harnessed the role of traditional circumcision, equipped healthcare facilities with tools for the provision of VMMC, and introduced VMMC camps to target young males.[9]

Despite these efforts, national VMMC coverage in South Africa remains far below the ambitious 80% coverage target. In latest estimates of national circumcision coverage from 2012, 47% of males 15 years and older in South Africa reported circumcision and the majority of these reported traditional circumcision (53%). There was, however, significant demographic and regional variations in circumcision coverage.[15,16] Circumcision coverage was lower among younger males 15–24 years (43%) and especially low in the KwaZulu-Natal region (23%), which has the highest regional HIV prevalence (17%).[15] In the 19^th^ century, the predominant culture in the KwaZulu-Natal region, the Zulu culture, stopped practicing traditional circumcision to retain warrior-aged males; [17] in 2012, 68% of circumcised males in this region reported medical male circumcisions.[15]

A number of studies in sub-Saharan Africa have examined factors associated with circumcision prevalence and found younger age, Muslim faith, urban residence, higher education, and wealth to be positively associated with the prevalence of circumcision.[18–25] A major limitation of these studies, however, was that they used cross-sectional data and thus included males who were circumcised for religious or cultural reasons prior to the known benefits of circumcision on reduced HIV transmission.[18–25] Understanding what individual characteristics predict VMMC incidence during an era of known circumcision benefits is important so that policy makers can understand both whom existing VMMC campaigns are reaching and the form future VMMC campaigns must take to expand their reach. To our knowledge, there are no empirical estimates of VMMC incidence or analyses of what individual characteristics predict VMMC incidence in a real-world, sub-Saharan African population during an era of known circumcision benefits.

We used longitudinal, population-based surveillance data from KwaZulu-Natal, South Africa to measure VMMC prevalence, incidence and predictors of VMMC incidence in a high HIV prevalence, real-world population. Such knowledge is crucial for tailoring existing VMMC campaigns and informing future VMMC campaigns in South Africa.

## Methods

### Study setting

The Africa Health Research Institute (AHRI) demographic surveillance site (438-km^2^) in the rural uMkhanyakude district of KwaZulu-Natal, South Africa has been collecting longitudinal surveillance data since 2003.[26] In 2014, 34% of all adults and 20% of all males 15 to 49 years in the AHRI data were living with HIV.[27] From 2009 to 2011, HIV incidence among males 15 to 49 years in the AHRI data was 4.3/100 person-years (PY).[27] In 2003, only 6% of males 15 to 49 in the AHRI data reported being circumcised. While this region is traditionally non-circumcising, in 2009 the current Zulu king, King Goodwill Zwelithini, reintroduced the practice of male circumcision to prevent HIV transmission.[28–30]

The AHRI surveillance collects sociodemographic, health and sexual behavior data through annual surveys. All household members 15 years and older are invited to participate in individual face-to-face interviews with research assistants and complete HIV testing, which is done through the collection of dried blood spots that are later tested for HIV. In the early survey years, participants were provided their HIV test results upon request. However, in later survey years (including the whole of the study period for this analysis), participants were not given their HIV test results and were instead referred to facility-based HIV testing and counseling services if they wanted to learn their HIV status. AHRI does not promote or provide VMMC as part of their surveillance. Circumcision-specific questions were included in the initial 2003 survey and then re-introduced in 2009.

Ethical approval for AHRI surveillance was granted by the Biomedical Research Ethics Committee, University of KwaZulu-Natal. This analysis was exempted from additional review by the Harvard T.H. Chan School of Public Health’s Institutional Review Board since it used anonymized secondary data.

### Participants and procedures

We included data collected between January 2009 and November 2014 for our analysis. To be included in our analyses, respondents had to be: (i) male; (ii) 15–49 years old (target circumcision age group); and (iii) a resident in the surveillance area. We constructed an open circumcision incidence cohort. Eligible respondents entered the cohort when they first reported their circumcision status. To be included in our circumcision incidence cohort, respondents had to additionally: (iv) self-report their circumcision status at least twice (thus, those entering the surveillance data in the last year of observation, 2014, were not included); and (v) self-report not being circumcised when they first appeared in the surveillance data.

### Circumcision status

We assumed that participants remained circumcised in all years following their first report of being circumcised and additionally assumed that respondents’ date of circumcision occurred midway between their last report of not being circumcised and first report of being circumcised. AHRI research assistants did not define circumcision to surveillance participants, thus circumcision status was determined by the participants themselves and may have included traditional circumcision. Respondents who reported being circumcised were asked why they got circumcised (i.e., cultural or health reasons) and where the circumcision was conducted (i.e., government hospital, private hospital, or VMMC camp). In KwaZulu-Natal, VMMC camps are two day events that often take place at schools during the holidays; here young males are taught life skills, undergo HIV counseling and testing, and are circumcised.[31]

#### Predictors

We considered a range of potential predictors of VMMC incidence.[18,19] At the individual level, we included participants’ age (15 to 19 years, 20 to 24 years, 25 to 29 years, 30 to 39 years, and 40 to 49 years), educational attainment (no education, primary education [1–7 years], secondary education [8–12 years], tertiary education [>12 years]), whether the respondent reported ever having had sex, self-reported knowledge of HIV status (know, do not know–status not specified), and biological HIV status (determined from the dried blood spots collected during surveillance). We assumed the date of HIV seroconversion for respondents living with HIV to be midway between a respondent’s last negative and first positive biological HIV test. We imputed participant’s age when it was missing from other non-missing observations and adjusted participant’s biological HIV status to negative for all years preceding the estimated seroconversion date and positive for all years following the estimated seroconversion date. At the household level, we included a household asset index (quintiles), distance to nearest clinic (kilometers), and urbanicity (rural or peri-urban/urban). We derived our household asset index from a principal components analysis of 28 binary indicators for household items owned; [32–34] this index has been used in many other analyses that use AHRI data.[35,36]

### Statistical methods

We calculated circumcision prevalence by dividing the number of males who self-reported being circumcised by all males in the surveillance data who reported their circumcision status for each year and age group; we calculated confidence intervals using binomial confidence limits. We calculated annualized percentage change in circumcision prevalence over time by dividing the difference in circumcision prevalence from 2014 to 2009 by circumcision prevalence in 2014 and the number of years of observation, then multiplying by 100. We calculated circumcision incidence for a smaller subset of the sample that met the inclusion criteria for the open circumcision incidence cohort. We computed circumcision incidence for all participants and for specific age groups (based on cohort entry) using males that we identified as newly circumcised and person-years of follow-up; we used Poisson confidence limits to calculate confidence intervals.[37]

We used multivariable survival models with Weibull distributions to identify significant predictors of circumcision incidence, using covariate values from the year participants entered the data (i.e., baseline, non-time-varying) and robust standard errors. We explored using Cox proportional hazard models, but our data violated the proportional hazards assumption (see S1, S2, S3, S4, S5 and S6 Figs).[38] We began with bivariate models to understand whom circumcision campaigns were reaching, and then included all covariates in a multivariable model. We used the missing indicator method to account for missing covariates data in all models.[38]

We conducted four sensitivity analyses to test the robustness of the results from the multivariable model: (1) a logistic regression model, where we used baseline characteristics to predict circumcision incidence (no person-years); (2) a survival model, where we assumed that all males with inconsistent self-reported circumcision status (i.e. reported being uncircumcised after reporting being circumcised) remained uncircumcised; (3) a survival model, where we used multiple imputation chained equations (MICE) for missing data; and (4) a survival model, where we dropped all missing data. We used MICE to impute the predictor variables that remained missing after the adjustments for missing data described above; we used predictive mean matching for continuous variables (distance to the nearest clinic), logistic regression for binary variables (ever had sex, knowledge of HIV status, biological HIV status), ordered regression for ordered categorical variables (asset index), and multinomial regression for non-ordered categorical variables (urbanicity, education). We performed MICE using the ‘mi impute’ procedure in Stata 13.1 (Stata Corporation, College Station, Texas).[39] We used Weibull distributions for all sensitivity analyses that used survival models (see S2 Table). We also conducted a stratified analysis that stratified participants by biological HIV status and explored the predictors of circumcision incidence among biologically confirmed HIV-negative and HIV-positive males (see S3 Table).

We performed all statistical analyses using Stata 13.1.

## Results

**Fig 1:**
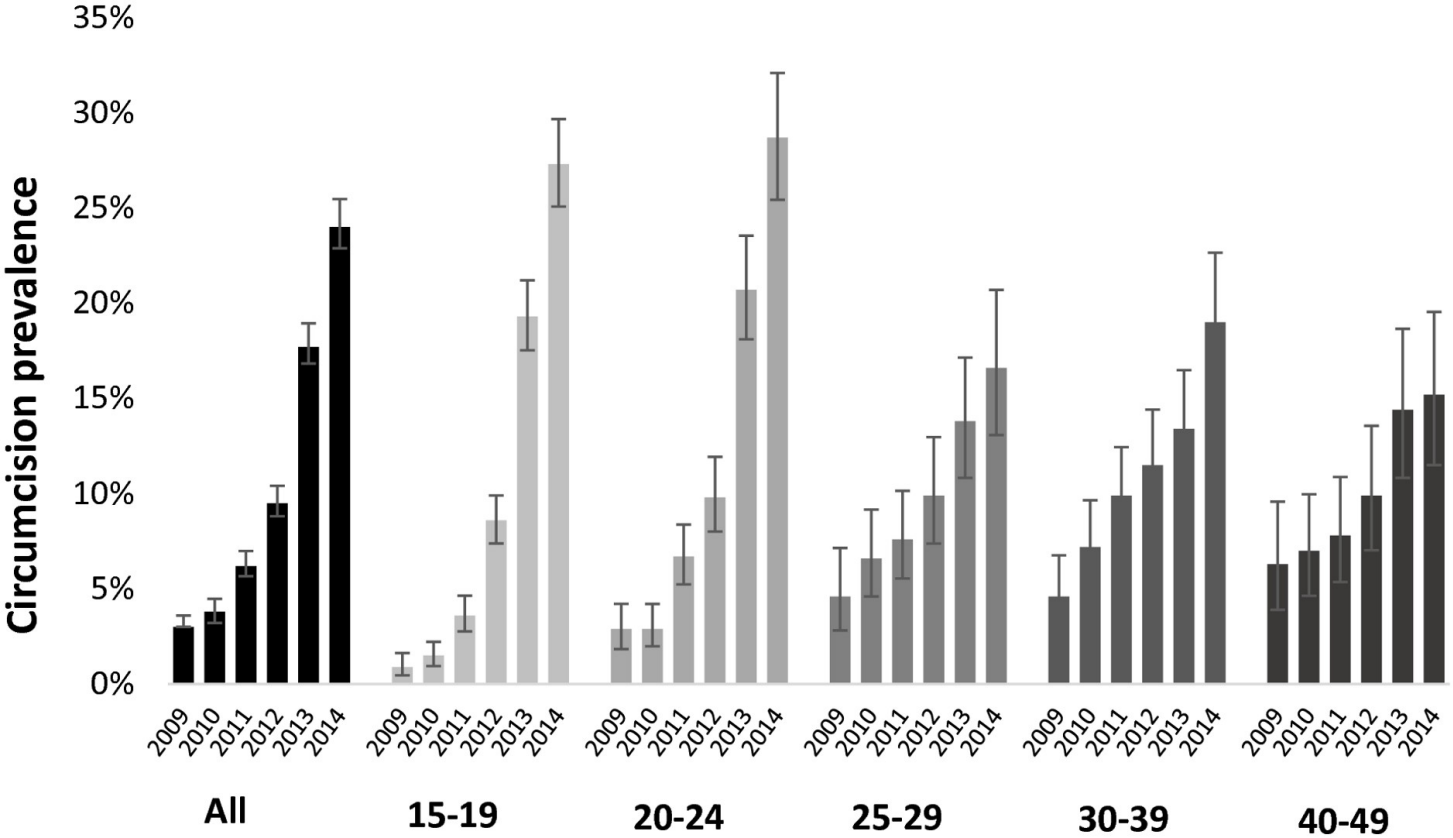
Circumcision prevalence over time and age groups, AHRI surveillance data: 2009–2014.

Between January 2009 and November 2014, 13,182 unique males 15–49 years were sampled, 13,022 reported their circumcision status, and 1,759 of these males (14%, 95% CI 13–14%) self-reported ever being circumcised. As shown in Fig 1, self-reported population circumcision prevalence for males 15 to 49 years in the surveillance data rose from 3% (95% CI: 2–4%) in 2009 to 24% (95% CI 23–25%) in 2014, a 140% annualized percent change. The change in circumcision prevalence was particularly large among males in the youngest age group, 15–19 years; from 2009 to 2014 circumcision prevalence in this age group rose from 1% (95% CI 0–2%) to 27% (95% CI 25–30%), a 26-fold increase.

**Fig 2:**
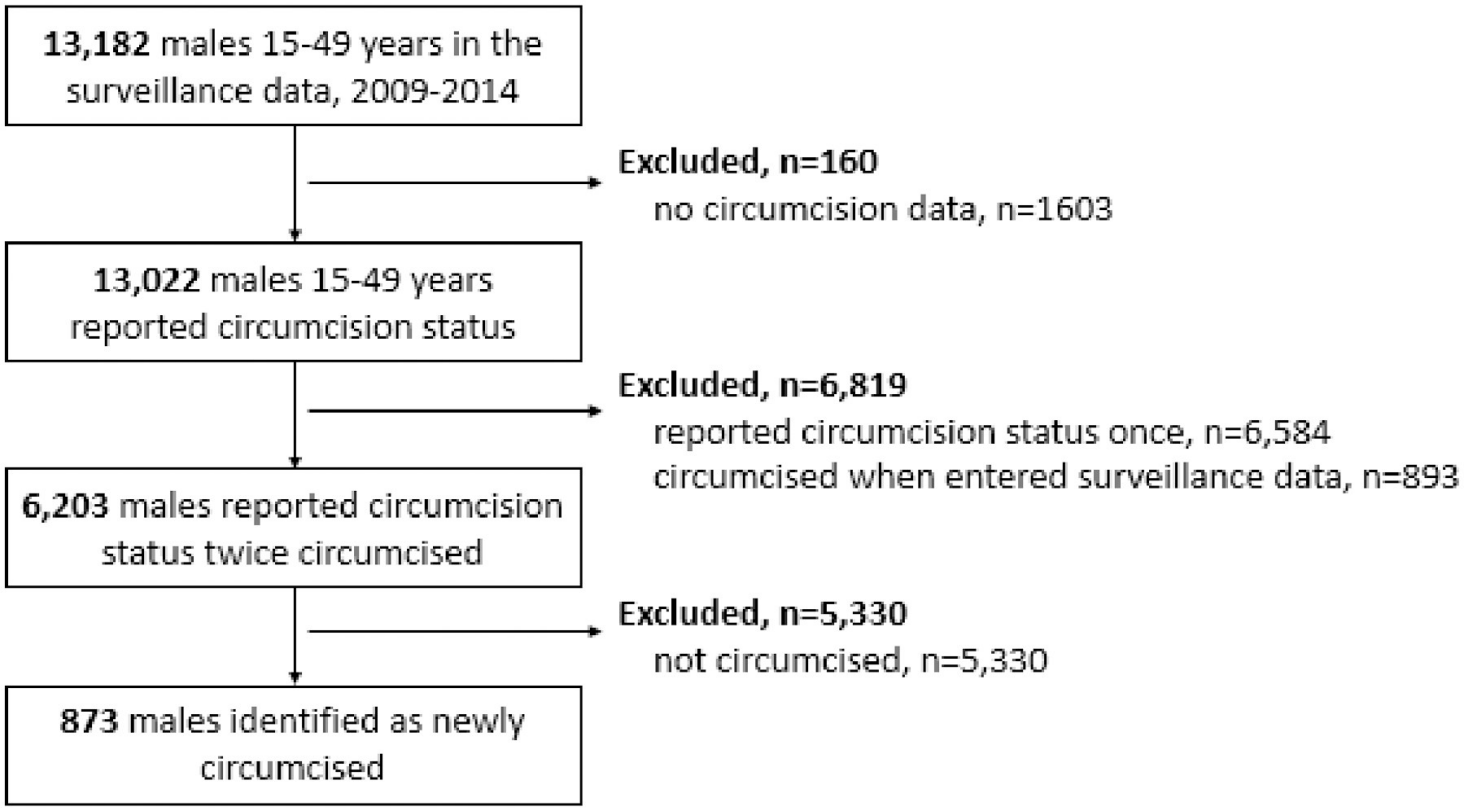
AHRI surveillance participants included in our circumcision incidence cohort.

Of all the 13,022 males who reported their circumcision status, 6,203 meet the inclusion criteria for our open circumcision incidence cohort, Fig 2. Males in this cohort self-reported their circumcision status between two and six times (59.9% twice; 27.0% three times; 10.0% four; 2.6% five; 0.6% six). Due to the open nature of the cohort, males often joined and left the surveillance data. The median gap in self-reported circumcision status was 374 days (interquartile range 353 to 699 days); 25% of gaps were less than one year apart, 25% were one to two years apart, and 50% were greater than two years.

Table 1 describes baseline descriptive characteristics of the males in the open circumcision incidence cohort. The majority of the males in this cohort were young (52.3% were 15–19 years old), had some secondary education, lived in a peri-urban or urban setting, did not know their HIV status, and were biologically confirmed to be HIV-negative. These characteristics reflect the socio-demographics of this area, including its population pyramid and socioeconomic status.[26] The socio-demographic characteristics of the males who did not meet the inclusion criteria did not differ from those included in the sample (see S1 Table).

**Table 1:** Baseline characteristics of participants in the VMMC incidence cohort^1^.

Characteristic		Respondents, n (%)*
Year entered cohort:	*2009*	2078 (33.5)
	*2010*	1697 (27.4)
	*2011*	961 (15.5)
	*2012*	1021 (16.5)
	*2013*	446 (7.2)
Age category:	*15–19*	3242 (52.3)
	*20–24*	1130 (18.2)
	*25–29*	594 (9.6)
	*30–39*	733 (11.8)
	*40–49*	504 (8.2)
Education:	*No education*	102 (1.7)
	*Primary (1–7 years)*	1203 (20.1)
	*Secondary (8–12 years)*	4649 (77.6)
	*Tertiary (>12 years)*	34 (0.6)
Asset Index:	*Most deprived*	1091 (20.0)
	*2*^*nd*^ *most deprived*	1089 (20.0)
	*Middle*	1090 (20.0)
	*2*^*nd*^ *least deprived*	1090 (20.0)
	*Least deprived*	1090 (20.0)
Urbanicity:	*Rural*	1743 (29.6)
	*Peri-urban or urban*	4137 (70.4)
	Distance to nearest clinic, median (km):	2.6
Ever had sex:	*Yes*	2614 (49.8)
	*No*	2634 (50.2)
Know HIV status:	*Yes*	2480 (41.0)
	*No*	3569 (59.0)
HIV status:^†^	*HIV-positive*	478 (10.2)
	*HIV-negative*	4201 (89.8)
Subjects (n)		6,203

*data represent % of respondents unless otherwise specified.

^†^ Biologically confirmed. Km: Kilometers.

^1^ The differences in the number of participants who reported each variable are attributable to variations in completeness of variable reporting. There is greater incompleteness for the ‘ever had sex’ and ‘HIV status’ variables because the section of the survey in which these questions were asked requiring an additional consent process

**Fig 3:**
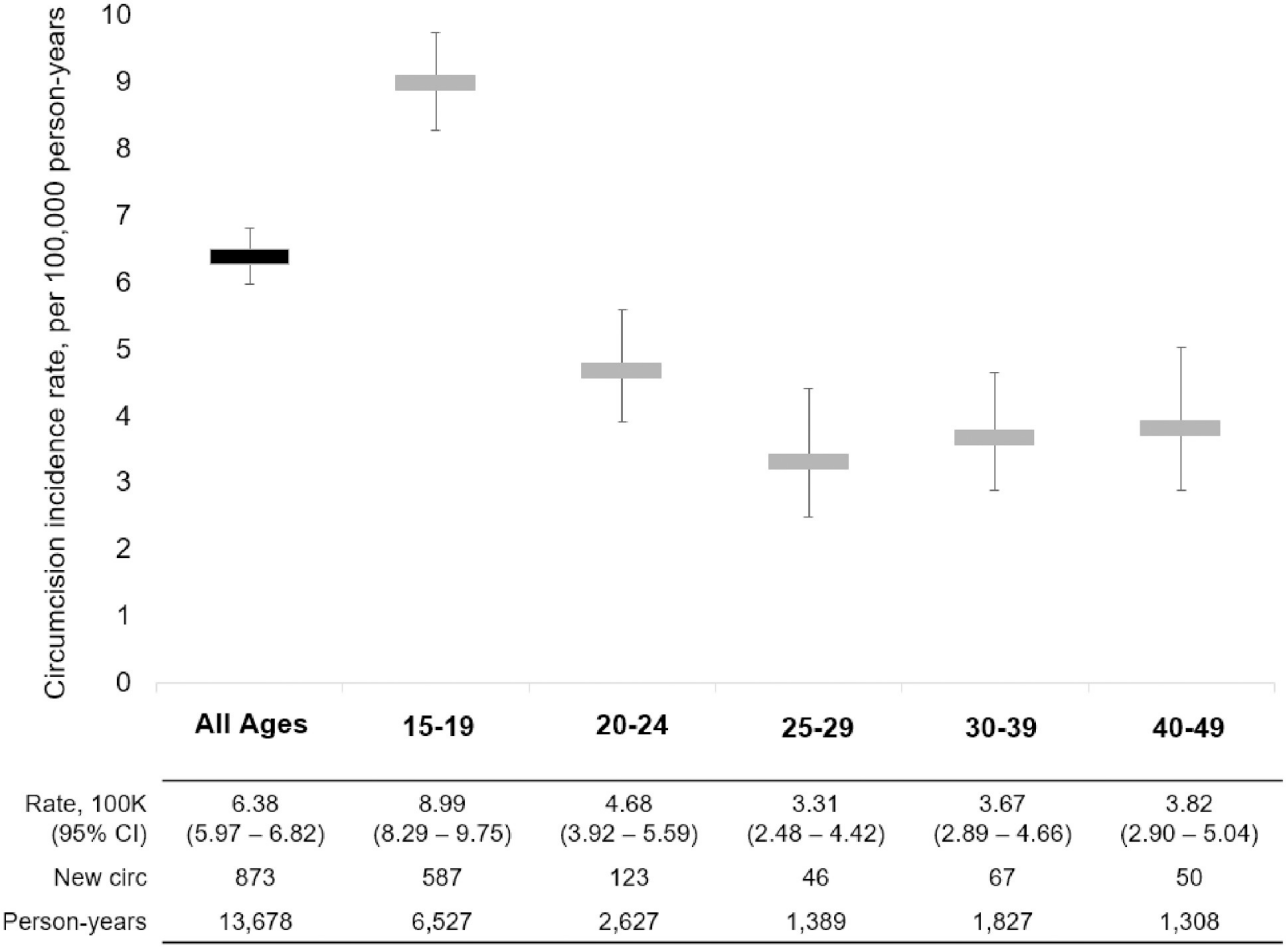
Circumcision incidence (with 95% CI) by age group, AHRI surveillance data: 2009–2014.

Fig 3 shows the circumcision incidence for all males in this cohort and males within specific age groups. From 2009 to 2014 we observed 873 new circumcisions over 13,678 person-years (PY) of follow-up, which translates into an overall circumcision incidence of 6.38/100 PY (95% CI 5.97–6.82/100 PY). There was, however, significant variation in circumcision incidence by age group; circumcision incidence was highest for the males 15–19 years (8.99/100 PY, 95% CI 8.29–9.75/100 PY), and consistently lower across older age groups.

The bivariate and multivariable associations with circumcision incidence are shown in Table 2. In the multivariable survival analysis age, household distance to a healthcare facility, self-reported knowledge of HIV status, and biologically confirmed HIV status were the strongest predictors of circumcision incidence. Circumcision incidence was half as likely in all ten-year age groups 20 years and older compared to the reference age group of 15 to 19 years. The likelihood of circumcision incidence decreased for each kilometer increase in household distance to the nearest healthcare facility (adjusted hazard ratio [aHR] 0.90, 95% CI 0.85–0.94) and was higher among individuals who self-reported knowing their HIV status (aHR 1.28, 95% CI 1.11–1.49) and who were biologically confirmed HIV-negative (aHR 1.67, 95% CI 1.16–2.38). Self-reported knowledge of HIV status switched from being non-significant in the bivariate analysis to highly significant in the multivariable analysis. Very low education (none) was negatively associated with circumcision incidence, while very high education (tertiary) was positively associated with circumcision incidence in the multivariable analysis. Self-reported history of sexual activity, household urbanicity, and household assets were not significant predictors of circumcision incidence.

**Table 2:** Predictors of circumcision incidence, 2009–2014: Bivariate analyses and adjusted multivariable analysis^1^.

Dependent variable		Bivariate analyses^2^	Multivariable analysis
		HR (95% CI)	adj HR (95% CI)
Base year:	*2009*	*ref*	*ref*
	*2010*	1.90 (1.58–2.29)	1.81 (1.50–2.18)
	*2011*	2.84 (2.31–3.50)	2.69 (2.18–3.32)
	*2012*	5.65 (4.62–6.92)	5.07 (4.10–6.29)
	*2013*	8.02 (5.98–10.78)	7.16 (5.28–9.71)
Age category:	*15–19*	*ref*	*ref*
	*20–24*	0.50 (0.41–0.61)	0.57 (0.45–0.71)
	*25–29*	0.35 (0.26–0.48)	0.39 (0.28–0.55)
	*30–39*	0.39 (0.30–0.50)	0.44 (0.32–0.60)
	*40–49*	0.40 (0.30–0.54)	0.48 (0.34–0.67)
Education:	*No education*	0.18 (0.06–0.55)	0.27 (0.08–0.87)
	*Primary (1–7)*	1.07 (0.91–1.26)	0.93 (0.79–1.11)
	*Secondary (8–12)*	*ref*	*ref*
	*Tertiary*	1.55 (0.72–3.35)	2.63 (1.17–5.89)
Asset Index:	*Lowest quintile*	*ref*	*ref*
	*2*^*nd*^ *lowest quintile*	0.93 (0.73–1.18)	0.82 (0.64–1.05)
	*Middle quintile*	1.22 (0.97–1.53)	0.96 (0.76–1.22)
	*2*^*nd*^ *highest quintile*	1.43 (1.14–1.79)	1.02 (0.80–1.29)
	*Highest quintile*	1.49 (1.19–1.87)	1.04 (0.81–1.32)
Peri-urban or urban:		0.86 (0.75–1.00)	0.94 (0.80–1.09)
	Distance to nearest healthcare facility (km):	0.91 (0.88–0.95)	0.90 (0.85–0.94)
Ever had sex:		0.54 (0.47–0.63)	1.09 (0.91–1.31)
Know HIV status:		1.00 (0.87–1.14)	1.28 (1.11–1.49)
HIV-negative:^†^		2.33 (1.68–3.23)	1.67 (1.16–2.38)
Subjects (n):		6,203	6,203
New circumcisions (n):		873	873
Person-years of observation:		13,678	13,678
Akaike information criterion (AIC):		5722	5722

^†^Biologically confirmed. Km: Kilometers.

^1^Survival models with Weibull distributions.

^2^Each bivariate analysis had 873 incident circumcisions and 6,203 person-years of follow-up observation

All significant predictors of circumcision incidence remained significant in the sensitivity analyses with the exception of biologically confirmed HIV-negative status (see S2 Table). In the analysis that stratified males by biological HIV status (see S3 Table), all predictors except knowledge of HIV status remained significant among biologically confirmed HIV-negative males. While none of the predictors remained significant among HIV-positive males, due to small numbers, there were qualitatively different patterns of circumcision predictors. For example, knowledge of HIV status decreased circumcision incidence among biologically confirmed HIV-positive males, which makes sense because these individuals are not encouraged by the South African government to uptake VMMC for HIV prevention.[12–14]

**Fig 4:**
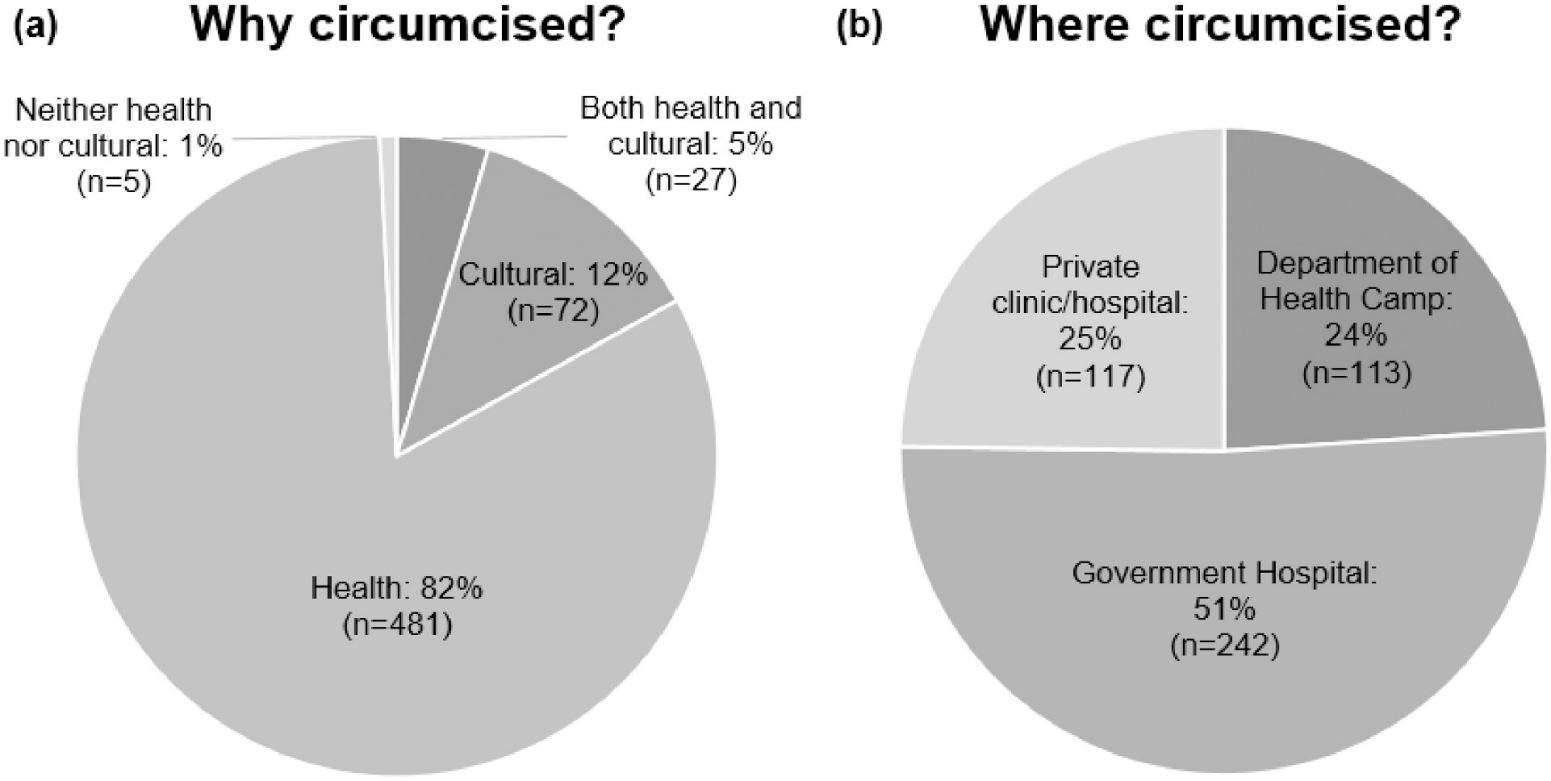
Newly circumcised males reporting (a) why and (b) where they were circumcised, AHRI surveillance data: 2009–2014.

Fig 4 shows why males who we identified as newly circumcised in the AHRI data got circumcised and where they went for circumcision. The majority of newly circumcised males reported getting circumcised for health reasons (82%, 481/585) as opposed to cultural reasons (12%, 72/585). The majority of newly circumcised males also reported getting circumcised at a government hospital (51%, 242/474); however, private clinics/hospitals (25%, 117/474) and KwaZulu-Natal Department of Health VMMC camps (24%, 113/474), [31] were also common locations for circumcision.

## Discussion

In a real-world, high HIV prevalence population in KwaZulu-Natal, South Africa during an era of known circumcision benefits on reduced HIV transmission (2009–2014), we found that younger age, proximity to the nearest healthcare facility, biologically confirmed HIV-negative status, and knowledge of HIV status were significant predictors of circumcision incidence. Within our circumcision incidence cohort, circumcision incidence rates were substantially higher for males 15 to 19 years compared to males of other age groups. The majority of circumcised males self-reported getting circumcised for health reasons and going to a government hospital for circumcision. Despite impressive gains in circumcision prevalence over this study period, in 2014 circumcision remained far below South Africa’s 80% national VMMC prevalence target: prevalence was below 30% in all age groups, even in the younger age groups where the gains in circumcision incidence were largest.[9,11]

Circumcision prevalence in this rural population in KwaZulu-Natal could be increasing for a number of reasons including changing government policies and social norms. We have discussed the South African government’s efforts to promote VMMC uptake through communication campaigns, equipping healthcare facilities with tools for VMMC expansion, and targeting youth with VMMC camps.[9] However, changing social norms could also have contributed to increases in VMMC prevalence in this region, including social pressures among peers, changes in ethnic (Zulu) expectations, increases in education, and overall modernization (i.e., education, age, access to technology, and wealth). Future research is necessary to disentangle which of these factors most significantly contributed to the observed changes.[40]

While younger males appear to be circumcising at a higher rate compared to older males in our study population, the incidence of the best known, one-time HIV prevention intervention among males 20 years and over remains low and their risk of HIV infection remains high. In this longitudinal, population-based surveillance data, the rate of circumcision incidence for all males aged 15 to 49 years in this population (6.4/100 PY from 2009 to 2014) is not much higher than the estimated HIV incidence rate in the same population (4.3/100 PY from 2009 to 2011).[27] Circumcision incidence rates were substantially higher than HIV incidence rates for 15 to 19 year olds; but the two rates were nearly equal for all older age groups.[27] The rapid, but as yet insufficient, rise in circumcision rates in a traditionally non-circumcising community suggests that further uptake is still required.

To achieve national circumcision targets, policy makers need to understand both whom circumcision campaigns are reaching and what factors predict circumcision incidence. Our bivariate analyses provide evidence on the first question, showing that in this population, circumcision incidence was greatest among males who were: younger; have some education; are from wealthier households; live in rural (compared to peri-urban or urban) areas; live closer to clinics; have never had sex; and are biologically confirmed HIV-negative. These findings highlight the need to expand circumcision opportunities for low-socioeconomic status, sexually experienced, somewhat older males.

Our multivariable analysis provides evidence to the question: what interventions are needed in low-circumcising groups? Our finding that living further from a healthcare facility remained a risk factor for not getting circumcised, after adjusting for other factors, suggests that mobile circumcision clinics might play an important role in increasing circumcision incidence–especially since the majority of new circumcisions in this population are self-reported to be in government hospitals. Biologically-confirmed HIV-negative status was also associated with increased circumcision incidence in our multivariable analysis. This reflects South African circumcision guidelines, which recommend the procedure only for males not living with HIV. While circumcision does not reduce onward transmission for HIV-positive males, careful messaging around additional circumcision health benefits (e.g. reduced genital ulcer disease) for males living with HIV may be important in raising circumcision rates in this setting.[41,42]

A key policy-relevant finding of our multivariable analysis is that self-reported knowledge of HIV status is a significant predictor of circumcision incidence. It is possible that males who know their HIV status have better access to health services or take advantage of health services at the point of circumcision. Alternatively, knowledge of HIV-negative status may persuade males in KwaZulu-Natal to undertake VMMC. Only 44.4% of males reported knowing their HIV status in AHRI surveillance in 2014, which presents an opportunity to increase VMMC by increasing knowledge of HIV status. Knowledge of HIV status might be increased through improved HIV testing and counseling services, including door-to-door home-based testing [43,44] or HIV self-testing.[45–50] These HIV testing interventions should be linked with VMMC referrals, including the provision of financial incentives for VMMC, [51] to additionally increase VMMC uptake.

This study has considerable strengths. This study is one of few to measure circumcision incidence in a sub-Saharan African settings, [52] and the first to evaluate predictors of circumcision incidence using population-representative longitudinal data from a WHO VMMC priority country. Our finding that younger compared to older age is associated with VMMC is consistent with other studies on this topic.[18–25]

Our findings, however, should be interpreted in the context of some limitations. First, given the open nature of this cohort, there were missing data. This missing data were notable for sexual behavior and biological HIV status data because participants could opt out of survey sections that collected this data. The robustness of results in the MICE and complete case sensitivity analyses, however, suggest that missing data were unlikely to have influenced our findings.

Second, our analyses relied on self-reported circumcision status and the definition of circumcision was not clarified to surveillance participants. Due to the rarity of circumcision within this population, some males might have been confused as to what circumcision was or may have confused partial traditional circumcision with medical circumcision; as indicated by some participants reporting not being circumcised in survey years following ones where they reported being circumcised. Self-reported circumcision status may have also been subject to social desirability bias in later years when circumcision promotion campaigns were more prevalent throughout KwaZulu-Natal. While self-reported circumcision status has not been validated in the AHRI data, a validation study conducted in Kenya found the accuracy of self-reported circumcision status to be 99.0%.[53] To account for any time-varying components of this bias, we included year-specific indicator variables in our analyses.

Third, our sample covered only those aged 15 years and over and as a result, these findings cannot be generalized to younger age groups. This is a limitation because South African national statistics suggest that 40% of all new circumcisions occur among males 10 to 14 years.[54] Finally, we did not exclude males infected with HIV from our analysis, and these males might have been more unlikely to uptake circumcision because they were not the target of VMMC demand creation campaigns in South Africa. None the less, we purposely included HIV-positive males in our analysis to understand if VMMC campaigns were targeting those most likely to benefit from the intervention.

## Conclusion

Increasing VMMC in a traditionally non-circumcision population is a challenge; the challenge is particularly daunting in the light of South Africa’s target to achieve national 80% circumcision coverage among males 15 to 49 years.[9,11] Since 2009, the prevalence of circumcision among males in KwaZulu-Natal has risen dramatically as a result of efforts by the South African government, however, a significant gap between current and desired circumcision coverage remains. Our analyses shows that current VMMC efforts in KwaZulu-Natal appear to be effectively targeting young males, but that distance to healthcare facilities and knowledge of HIV status remain important barriers to VMMC uptake. In this setting and in similar settings, policy makers should explore the use of mobile circumcision clinics and the expansion of existing and innovative HIV testing strategies to increase VMMC incidence. Circumcision is a powerful HIV prevention tool; understanding ways to more effectively target VMMC interventions to slow the HIV incidence in a high HIV prevalence population is a valuable contribution towards a future AIDS-free generation.[55]

## Supporting information

S1 Fig Kaplan-Meier survival estimates of circumcision incidence by base year, 2009–2014. (TIFF)

S2 Fig Kaplan-Meier survival estimates of circumcision incidence by age group, 2009–2014. (TIFF)

S3 Fig Kaplan-Meier survival estimates of circumcision incidence by education, 2009–2014. (TIFF)

S4 Fig Kaplan-Meier survival estimates of circumcision incidence by distance (km) to the nearest health clinic (quartiles), 2009–2014. (TIFF)

S5 Fig Kaplan-Meier survival estimates of circumcision incidence by HIV status knowledge, 2009–2014. (TIFF)

S6 Fig Kaplan-Meier survival estimates of circumcision incidence by HIV status, 2009–2014. (TIFF)

S1 Table Baseline characteristics of participants 15–49 years not included in the VMMC incidence cohort. (DOCX)

S2 Table Predicators of circumcision incidence, 2009–2014: Sensitivity analyses. (DOCX)

S3 Table Predictors of circumcision incidence, 2009–2014: Stratified analysis by biological HIV status. (DOCX)
