# Supplementary material for: Predictors of male circumcision incidence in a traditionally non-circumcising South African population-based cohort
Source: PLoS One. 2018 Dec 19;13(12):e0209172. doi: 10.1371/journal.pone.0209172 (PMC6300268; doi:10.1371/journal.pone.0209172)
Supplement: S1 Table — (DOCX) [file pone.0209172.s007.docx]

| Characteristic | Respondents, n (%)* |
| --- | --- |
| Year entered cohort: *2009* | 1225 (17.6) |
| *2010* | 1213 (17.4) |
| *2011* | 1141 (16.4) |
| *2012* | 1106 (15.9) |
| *2013* | 1163 (16.7) |
| *2014* | 1131 (16.2) |
| Age category: *15-19* | 2650 (38.0) |
| *20-24* | 1443 (20.7) |
| *25-29* | 987 (14.1) |
| *30-39* | 1256 (18.0) |
| *40-49* | 643 (9.2) |
| Education: *No education* | 125 (1.7) |
| *Primary (1-7)* | 884 (12.7) |
| *Secondary (8-12)* | 4729 (67.8) |
| *Tertiary* | 56 (0.8) |
| *Missing* | 1185 (17.0) |
| Asset Index: *Most deprived* | 1092 (15.6) |
| *2^nd^ most deprived* | 1109 (15.9) |
| *Middle* | 1084 (15.5) |
| *2^nd^ least deprived* | 1191 (17.1) |
| *Least deprived* | 1284 (18.4) |
| *Missing* | 1219 (17.5) |
| Urbanicity: *Rural* | 1680 (27.1) |
| *Peri-urban or urban* | 4016 (64.7) |
| *Missing* | 507 (8.2) |
| Distance to nearest clinic, median (km): | 2.5 |
| Ever had sex:  *Yes* | 2222 (32.8) |
| *No* | 3188 (45.7) |
| *Missing^1^* | 1569 (22.5) |
| Know HIV status: *Yes* | 3228 (46.3) |
| *No* | 3574 (51.2) |
| *Missing* | 177 (2.5) |
| HIV status:^†^ *HIV-positive* | 719 (10.3) |
| *HIV-negative* | 2884 (41.3) |
| *Missing^1^* | 3376 (48.4) |
| Subjects (n) | 6,979 |

**S1 Table. Baseline characteristics of participants 15-49 years not included in the VMMC incidence cohort**

* data represent % of respondents unless otherwise specified.

† Biologically confirmed. Km: Kilometers.

^1^ The percentage of missing data for ‘ever had sex’ and ‘HIV status’ questions is large because these sections of the survey requiring an additional consent process.
