# Supplementary material for: Predictors of male circumcision incidence in a traditionally non-circumcising South African population-based cohort
Source: PLoS One. 2018 Dec 19;13(12):e0209172. doi: 10.1371/journal.pone.0209172 (PMC6300268; doi:10.1371/journal.pone.0209172)
Supplement: S2 Table — (DOCX) [file pone.0209172.s008.docx]

|  | (1) Logistic reg^1^ | (2) Survival^2^: circ error | (3) Survival^3^: MICE | (4) Survival^4^: no miss |  |
| --- | --- | --- | --- | --- | --- |
| Dependent variable | adj OR (95% CI) | adj HR (95% CI) | adj HR (95% CI) | adj HR (95% CI) |  |
| Base year: *2009* | *ref* | *ref* | *ref* | *ref* |  |
| *2010* | 1.29 (1.06 - 1.59) | 1.81 (1.42 - 2.31) | 1.82 (1.52 - 2.20) | 1.94 (1.45 - 2.61) |  |
| *2011* | 1.51 (1.20 - 1.90) | 3.10 (2.38 - 4.04) | 2.69 (2.16 - 3.32) | 3.20 (2.30 - 4.44) |  |
| *2012* | 1.66 (1.32 - 2.08) | 5.71 (4.41 - 7.40) | 5.00 (4.06 – 6.17) | 5.30 (3.83 - 7.34) |  |
| *2013* | 1.21 (0.89 - 1.66) | 10.82 (7.76 - 15.08) | 7.24 (5.37 – 9.78) | 11.72 (7.81 - 17.60) |  |
|  |  |  |  |  |  |
| Age category: *15-19* | *ref* | *ref* | *ref* | *ref* |  |
| *20-24* | 0.56 (0.44 - 0.72) | 0.51 (0.38 - 0.68) | 0.58 (0.46 - 0.72) | 0.45 (0.31 - 0.65) |  |
| *25-29* | 0.40 (0.28 - 0.56) | 0.30 (0.19 - 0.48) | 0.39 (0.28 - 0.54) | 0.33 (0.17 - 0.63) |  |
| *30-39* | 0.49 (0.36 - 0.68) | 0.37 (0.25 - 0.56) | 0.44 (0.32 - 0.60) | 0.27 (0.14 - 0.53) |  |
| *40-49* | 0.54 (0.37 - 0.78) | 0.42 (0.27 - 0.67) | 0.50 (0.35 - 0.72) | 0.32 (0.15 - 0.70) |  |
|  |  |  |  |  |  |
| Education: *No education* | 0.25 (0.08 - 0.80) | 0.47 (0.14 - 1.55) | 0.29 (0.09 - 0.95) | 0.81 (0.17 - 3.72) |  |
| *Primary (1-7)* | 1.11 (0.91 - 1.33) | 0.90 (0.73 - 1.11) | 0.94 (0.79 - 1.12) | 0.96 (0.75 - 1.24) |  |
| *Secondary (8-12)* | *ref* | *ref* | *ref* | *ref* |  |
| *Tertiary* | 2.82 (1.23 - 6.44) | 4.09 (1.66 – 10.11) | 2.64 (1.19 - 5.93) | 0.00 (0.00 - 0.00) |  |
|  |  |  |  |  |  |
| Asset Index: *Lowest quint.* | *ref* | *ref* | *ref* | *ref* |  |
| *2^nd^ lowest quintile* | 0.84 (0.64 - 1.09) | 0.87 (0.64 - 1.16) | 0.80 (0.64 - 1.01) | 0.81 (0.57 - 1.14) |  |
| *Middle quintile* | 1.05 (0.81 - 1.36) | 0.91 (0.68 - 1.22) | 0.94 (0.75 - 1.19) | 0.83 (0.58 - 1.17) |  |
| *2^nd^ highest quintile* | 1.04 (0.80 - 1.36) | 0.99 (0.74 - 1.33) | 0.95 (0.75 - 1.21) | 1.05 (0.74 - 1.47) |  |
| *Highest quintile* | 1.12 (0.86 - 1.45) | 0.95 (0.70 - 1.29) | 0.96 (0.76 - 1.22) | 0.92 (0.64 - 1.33) |  |
|  |  |  |  |  |  |
| Peri-urban or urban: | 0.91 (0.77 - 1.08) | 0.98 (0.81 - 1.19) | 0.93 (0.79 - 1.08) | 1.09 (0.86 - 1.37) |  |
|  |  |  |  |  |  |
| Distance to nearest clinic (km): | 0.91 (0.87 - 0.96) | 0.91 (0.86 - 0.96) | 0.90 (0.86 - 0.94) | 0.87 (0.81 - 0.94) |  |
|  |  |  |  |  |  |
| Ever had sex: | 1.08 (0.89 - 1.32) | 1.09 (0.87 - 1.36) | 1.04 (0.87 - 1.26) | 1.13 (0.87 - 1.46) |  |
|  |  |  |  |  |  |
| Know HIV status: | 1.28 (1.09 - 1.50) | 1.21 (1.01 - 1.46) | 1.27 (1.09 - 1.46) | 1.31 (1.04 - 1.64) |  |
|  |  |  |  |  |  |
| HIV-negative:^†^ | 1.71 (1.19 - 2.48) | 1.53 (0.97 - 2.41) | 1.46 (1.01 – 2.12) | 1.52 (0.82 - 2.85) |  |
|  |  |  |  |  |  |
| Subjects (n): | 6,203 | 6,203 | 6,203 | 3,183 |  |
| New circumcisions (n): | 873 | 873 | 873 | 377 |  |
| Person-years of observation: | n/a | 13,678 | 13,678 | 7,274 |  |
| Akaike information criterion (AIC): | 4870 | 4211 |  | 2631 |  |

**S2 Table. Predictors of circumcision incidence, 2009-2014: Sensitivity analyses**

Km: Kilometers. Circ: circumcision. Reg: regression. † Biologically confirmed

^1^Missing indicator (MI) for missing data, no person-years because logistic regression model; ^2^Weibull distribution, all men with errors in reported circumcision status over time considered never circumcised; ^3^Weibull distribution, multiple chained equations (MICE) for missing data; ^4^Weibull distribution, complete case analysis.
