# Supplementary material for: Predictors of male circumcision incidence in a traditionally non-circumcising South African population-based cohort
Source: PLoS One. 2018 Dec 19;13(12):e0209172. doi: 10.1371/journal.pone.0209172 (PMC6300268; doi:10.1371/journal.pone.0209172)
Supplement: S3 Table — (DOCX) [file pone.0209172.s009.docx]

|  | HIV-negative men^1^ | HIV-positive men^2^ |  |
| --- | --- | --- | --- |
| Dependent variable | adj HR (95% CI) | adj HR (95% CI) |  |
| Base year: *2009* | *ref* | *ref* |  |
| *2010* | 1.99 (1.50 - 2.63) | 1.78 (0.41 - 7.74) |  |
| *2011* | 3.31 (2.45 - 4.48) | 0.72 (0.05 - 11.22) |  |
| *2012* | 5.59 (4.16 - 7.51) | 2.56 (0.34 - 19.26) |  |
| *2013* | 11.1 (7.60 - 16.33) | 3.49 (0.40 - 30.32) |  |
|  |  |  |  |
| Age category: *15-19* | *ref* | *ref* |  |
| *20-24* | 0.52 (0.37 - 0.72) | 0.23 (0.01 - 7.95) |  |
| *25-29* | 0.27 (0.15 - 0.49) | 0.29 (0.04 - 2.01) |  |
| *30-39* | 0.27 (0.15 - 0.49) | 0.51 (0.11v2.44) |  |
| *40-49* | 0.30 (0.16 - 0.59) | 0.24 (0.02 - 3.75) |  |
|  |  |  |  |
| Education: *No education* | 0.68 (0.15 - 3.07) | 0.00 (0.00 - 0.00) |  |
| *Primary (1-7)* | 0.92 (0.73 - 1.16) | 0.39 (0.06 - 2.75) |  |
| *Secondary (8-12)* | *ref* | *ref* |  |
| *Tertiary* | 1.41 (0.19 - 10.31) | -- |  |
|  |  |  |  |
| Asset Index: *Lowest quint.* | *ref* | *ref* |  |
| *2^nd^ lowest quintile* | 0.92 (0.67 - 1.25) | 0.37 (0.04 - 3.97) |  |
| *Middle quintile* | 0.90 (0.65 - 1.24) | 0.94 (0.14 - 6.19) |  |
| *2^nd^ highest quintile* | 1.07 (0.78 - 1.46) | 0.18 (0.02 - 1.84) |  |
| *Highest quintile* | 0.94 (0.67 - 1.31) | 0.00 (0.00 - 0.00) |  |
|  |  |  |  |
| Peri-urban or urban: | 1.02 (0.82 - 1.26) | 2.70 (0.38 - 19.25) |  |
|  |  |  |  |
| Distance to nearest clinic (km): | 0.91 (0.85 - 0.97) | 1.05 (0.80 - 1.39) |  |
|  |  |  |  |
| Ever had sex: | 1.06 (0.83 - 1.35) | 0.45 (0.11 - 1.82) |  |
|  |  |  |  |
| Know HIV status: | 1.19 (0.96 - 1.47) | 0.61 (0.14 - 2.69) |  |
|  |  |  |  |
| Subjects (n): | 2,942 | 241 |  |
| New circumcisions (n): | 365 | 12 |  |
| Person-years of observation: | 6,660 | 614 |  |
| Akaike information criterion (AIC): | 2514 | 130 |  |

**S3 Table. Predictors of circumcision incidence, 2009-2014: Stratified analysis by biological HIV status**

Km: Kilometers. Circ: circumcision. Reg: regression. † Biologically confirmed

^1^Survival models with Weibull distributions – biological confirmed HIV-negative men only.

^2^Survival models with Weibull distributions – biological confirmed HIV-positive men only.
